# Hidden distress: a qualitative study of sociocultural barriers to mental health help-seeking among young people in Zimbabwe

**DOI:** 10.3389/fpubh.2026.1816545

**Published:** 2026-06-15

**Authors:** Rufaro Hamish Mushonga, Concilia Tarisai Bere, Takudzwa Joylyn Mtisi, Jermaine Dambi, Owen Nyamwanza, Arnold Maramba, James January

**Affiliations:** 1 Department of Mental Health, Faculty of Medicine and Health Sciences, University of Zimbabwe, Harare, Zimbabwe; 2 The Friendship Bench, Harare, Zimbabwe; 3 Department of Rehabilitation Sciences, Faculty of Medicine and Health Sciences, University of Zimbabwe, Harare, Zimbabwe; 4 Centre for Sexual Health and HIV/AIDS Research, Harare, Zimbabwe; 5 Department of Community Medicine, Faculty of Medicine and Health Sciences, University of Zimbabwe, Harare, Zimbabwe; 6 Department of Psychiatry and Mental Health, Kamuzu University of Health Sciences, Blantyre, Malawi

**Keywords:** barriers, help-seeking, mental health, young people, Zimbabwe

## Abstract

**Background:**

Sociocultural barriers are powerful forces that may shape how young people experience, interpret, and respond to psychological distress in Zimbabwe. For many youth (aged 15–24 years), symptoms of common mental disorders (CMDs) are navigated within social environments where disclosure risks labelling, social exclusion, and moral judgment. This stigma is rarely experienced in isolation; it intersects with pressures to protect family reputation, distrust in formal and informal support systems, and gendered expectations governing emotional expression. While existing research has documented individual barriers to mental health care, limited evidence examines how these sociocultural forces converge to shape help-seeking pathways among Zimbabwean youth. This qualitative study explored how sociocultural factors influence decisions to seek or avoid care among young people with lived experience of CMDs.

**Methods:**

A descriptive qualitative research design was employed. We utilised in-depth interviews and focus group discussions with young people aged 15–24 years with lived experience of CMDs, their caregivers, peer counsellors, lay health workers, and church leaders in Harare and Bindura, Zimbabwe. Purposive sampling was employed to recruit participants until data saturation was reached. All interviews were audiotaped and transcribed verbatim, and all the data were analysed using inductive thematic analysis.

**Findings:**

The analysis revealed that help-seeking decisions were shaped by four interconnected sociocultural barriers; pervasive stigma; profound distrust in community and institutional spaces; family reputation pressures that prioritise concealment over disclosure; and restrictive gender norms that equate emotional expression with weakness, particularly among young men. Participants described how these factors intersect to create a dynamic where mental health concerns remain hidden rather than addressed.

**Conclusion:**

Our findings demonstrate that effective mental health interventions for Zimbabwean youth must address the multifaceted sociocultural landscape in which help-seeking decisions are made. Programmes need to simultaneously combat stigma, build institutional trust, engage families in destigmatisation efforts, as well as challenge restrictive gender norms. These strategies offer the most promising pathway to closing the mental health treatment gap for young people in Zimbabwe.

## 1 Introduction

Globally, 10–20% of young people suffer from common mental disorders (CMDs) like depression and anxiety, which account for 16% of the disease burden in this age group (1, 2). In sub-Saharan Africa, where 15–24 year olds comprise 19.4% of the population (3), an estimated 27% live with depression and 30% with anxiety disorders (4). Despite this, mental healthcare access in low-resource African settings remains extremely limited. Service utilisation stands at only 14 visits per 100, 000 people annually versus the global average of 1,051 (5). This treatment gap is not merely a product of workforce or resource shortages, severe as they are, but is profoundly shaped by the interconnected sociocultural forces including stigma, gendered expectations, distrust in formal systems, and family reputation, which actively silence young people and block their pathways to care. Structural barriers certainly exist: severe workforce shortage (1.4 mental health workers per 100,000 compared to the global average of 9), poor mental health literacy, limited services, poor infrastructure, high costs among others (6). When available, treatments are often non-evidence based, relying on brief consultations or unintegrated traditional/spiritual healing, leading to high dropout rates (7, 8). Consequently, treatment receipt among Africans is among the world’s lowest.

In Zimbabwe, the situation is particularly acute. Roughly 27% of youth aged 15–24 experience depression, a likely underestimate (9). The treatment gap is staggering: Zimbabwe has only 18 psychiatrists for a population of 17 million, with most mental health services concentrated in urban hospitals (10). Poverty, economic crisis, unemployment, climate change, and the COVID-19 pandemic have further exacerbated mental health struggles among this vulnerable group (11–13). Yet many young Zimbabweans do not seek help. The consequences of untreated CMDs among youth are severe and well documented: poor academic performance and school dropout, increased risk of substance abuse, strained family relationships, reduced economic productivity, and elevated suicide risk (14–17). Notably, evidence based mental health interventions do exist. One model is the Friendship Bench (FB), a task-sharing cognitive-behavioural therapy (CBT) intervention delivered by trained lay health workers (known as “grandmothers”) and peer counsellors (“youth buddies”) on wooden benches in primary care clinics. Depression is identified using the Shona Symptom Questionnaire (SSQ-14), a locally validated 14-item screening tool for CMDs. Clients who screen positive receive between 6 and 8 sessions of problem-solving therapy (PST), with referral to a nurse or psychiatrist for more severe cases. The FB has demonstrated effectiveness for CMDs in Zimbabwe and has been scaled nationally (18–20).

However, most young people rarely present to primary care clinics where FB is located, often due to stigma, lack of awareness, privacy concerns in clinic settings among others (21). Other available care pathways include NGOs, private psychologists unaffordable to most, Schools Psychological Services within the Education Ministry, and traditional or spiritual leaders (22). Thus, even when services technically exist, sociocultural barriers prevent youth from accessing them. Understanding why requires examining the layered social worlds in which young Zimbabweans’ distress unfolds.

Stigma remains a significant factor shaping how mental illness is perceived and responded to. In many communities across Zimbabwe and the broader sub-Saharan region, psychological distress is often interpreted through moral, spiritual, or character-based lenses, leading to those affected being labelled as weak, morally troubled, or socially ostracised (23–26). For young people navigating identity formation in highly monitored social environments, the fear of stigma can be particularly restrictive (21). Worries about labels, ridicule, or social rejection often prompt youth to hide symptoms, normalise symptom presentation, or delay seeking help until their condition deteriorates (27). Family reputation amplifies this silence (28). In collectivist cultures, mental illness is often seen as a reflection of family, upbringing, or social status rather than an individual issue (28–30). Young people tend to hide their distress to protect family honour, avoiding shame or dishonour. Families might discourage seeking formal help due to fears of gossip, social rejection, or lasting damage to their reputation (31–33). This creates a conflict for youth between protecting themselves and remaining loyal to their family, as revealing their struggles might be viewed as betraying or exposing the family.

Distrust in formal mental health systems is another major barrier (21). Privacy worries are heightened in tight-knit communities, where staying anonymous is hard (34, 35). Young people often fear that sharing their information with healthcare providers might lead to it being disclosed to families, schools, or community members. The history of underfunded mental health services in many low-income areas, along with lack of youth-friendly options, weakens trust in these institutions (36). In such environments, silence can act as a shield protecting youth from perceived institutional, social, or relational dangers, even when mental health services are physically available (27, 37).

Gendered expectations influence how individuals express distress and determine which support-seeking behaviours are acceptable. Cultural norms of masculinity often emphasise stoicism, independence, and emotional restraint, which can discourage boys and young men from admitting vulnerability or seeking mental health support (38–40). As a result, help-seeking in young men is often delayed until crises occur. Young women, although sometimes more permitted to show emotional distress, face unique reputational risks. Disclosure might lead to moral judgment, victim-blaming, or assumptions of sexual or social impropriety, especially when mental illness is associated with perceptions of “waywardness” or relational transgression (41). Therefore, gender influences not only the willingness to seek help but also the social repercussions of doing so (42–45).

These themes do not operate in isolation but intersect to create layered ecology of silence where stigma reinforces family secrecy, distrust legitimises avoidance of services, and gender norms dictate acceptable emotional expression. Despite growing recognition of these barriers, existing literature has largely examined them in isolation rather than as interacting forces. Guided by ecological systems theory (46) and intersectionality (47), this study asks: How do sociocultural barriers shape help seeking decisions among young people who have completed mental health treatment for depression? Our findings aim to inform culturally appropriate, youth-centred mental health strategies in Zimbabwe and similar contexts.

## 2 Methods

### 2.1 Study and setting

This study employed a descriptive qualitative research design to explore sociocultural barriers influencing help-seeking for CMDS among young people in Zimbabwe. Data collection took place between April 2025 to July 2025 in Harare, Zimbabwe’s capital and Bindura, located about 80 km north east of Harare. These locations were deliberately selected to capture variations in the sociocultural contexts in which help-seeking behaviours occur, rather than for direct comparative analysis. In Harare, the study was conducted in high-density suburbs and factors such as high unemployment, urban stressors and poverty among others contribute to mental health challenges among youth. In Bindura, the study was conducted in a rural setting, where mental health issues among youth are shaped by distinct factors, including limited access to mental healthcare, agricultural and livelihood pressures, seasonal food insecurity, social isolation, and the influence of traditional norms and expectations. These rural specific stressors create unique psychosocial burdens that affect young people’s emotional wellbeing and help-seeking behaviours.

### 2.2 Inclusion and exclusion criteria

Four groups of participants were included in this study. First, young people aged between 15 and 24 years, were included if they had received care for depression through the Friendship Bench (FB). To ensure sufficient exposure to the local context, participants were required to have resided in either Bindura or Harare for a minimum of 12 months. These participants in this age group had to be willing and able to provide written informed consent; and for those ≤18 years, written assent was required alongside parental or guardian consent.

Second, youth buddies (peer counsellors aged 18–25 years) and lay health workers were eligible if they had actively provided mental health services to young people for at least 12 months. These individuals had to be aged 18 years or older and willing to give written informed consent for participation.

Third, caregivers were included if they were primary guardians of a young person who had sought mental health services through the FB. Eligible caregivers had to have been actively involved in the caregiving process for at least 12 months ensuring familiarity with the young person’s mental health experiences. All caregivers had to be 18 years or older and able to provide informed consent.

Last, church leaders were eligible if they held a recognised position of authority within their community or official designation within their church and had actively lived and worked in the community for at least 12 months. They were also required to be 18 years or older and willing to provide informed consent. Participants were excluded from the study if they did not meet the specified inclusion criteria for the respective group.

### 2.3 Sampling and recruitment

#### 2.3.1 Sampling and sample size

Purposive sampling was used to ensure that all participants could meaningfully contribute to the research questions. Young people were chosen for their direct experience navigating mental healthcare pathways; lay health workers and “youth buddies” for their frontline perspectives; and caregivers and religious leaders to capture family and community forces shaping help-seeking. This multi-stakeholder approach aimed to generate a rich, triangulated understanding of sociocultural barriers to mental healthcare for youth in Zimbabwe. Sample size was guided by the principle of data saturation, defined operationally as the point at which no new codes or themes emerged from successive interviews within a given participant group. Saturation was assessed through iterative analysis, where transcripts were coded incrementally. The stopping criterion was three consecutive interviews with no new codes and team consensus that saturation had been achieved. Saturation was reached at different points across groups: lay health workers (*n* = 19) after approximately 14 interviews; youth buddies (*n* = 10) after approximately 8 interviews; young people with CMDs (*n* = 11) after approximately 9 interviews. For caregivers (*n* = 2) and religious leaders (*n* = 2), saturation was not achieved due to recruitment difficulties. Recruiting caregivers proved challenging, as many were reluctant to participate, reflecting the very stigma and reputational concerns under investigation. This difficulty in itself informative rather than merely a methodological shortfall. The religious leaders, the exploratory nature of their inclusion meant saturation was never a realistic goal. Their insights were intended as illustrative not exhaustive. Overall, saturation across the three primary groups was achieved after approximately 31 interviews (excluding caregivers and religious leaders), with the final 9 confirming existing themes without new codes. The total sample was 44 participants.

#### 2.3.2 Recruitment

Recruitment was conducted primarily through the FB programme. Young people aged 15–24 years (*n* = 11) were eligible if they had received mental healthcare for depression through the FB. All 11 young participants had completed a full course of treatment at the FB, defined as completing 6–8 sessions and being discharged from the programme. Recruitment was limited to those who had finished treatment to ensure that participants could reflect on their entire help-seeking journey, including barriers encountered from initial contact through to completion or dropout, without the confounding influence of ongoing active treatment. CMD experience was established through programme records indicating screening and enrolment using the Shona Symptom Questionnaire (SSQ-14), a locally validated to for CMDs. No additional diagnostic verification was conducted, as the study focused on lived help-seeking experiences rather than clinical confirmation. Other participants included trained peer counsellors (“youth buddies”; *n* = 10) and lay health workers (*n* = 19). Peer counsellors and lay health workers were identified by their supervisors. Caregivers (*n* = 2) were recruited only with the permission of the young person who received mental health services. Religious leaders (*n* = 2) were selected based on their active role in providing counselling or spiritual guidance to young people in their communities. The two religious leaders were pastors from a Pentecostal church in Harare known for engaging with youth mental health issues. A detailed summary of participant characteristics in presented in Table 1.

**Table 1:** Demographic characteristics of participants.

Participant ID	Category	Age	Sex
CG1	Caregiver	46	F
CG2	Caregiver	45	F
CHW1	Lay Health Worker	46	F
CHW2	Lay Health Worker	42	F
CHW3	Lay Health Worker	54	F
CHW4	Lay Health Worker	65	F
CHW5	Lay Health Worker	79	F
CHW6	Lay Health Worker	73	F
CHW7	Lay Health Worker	54	F
CHW8	Lay Health Worker	74	F
CHW9	Lay Health Worker	73	F
CHW10	Lay Health Worker	45	F
FBB01	Youth Buddy	20	F
FBB02	Youth Buddy	22	F
FBB03	Youth Buddy	21	M
FBB04	Youth Buddy	20	F
FBB05	Youth Buddy	23	F
FBB06	Youth Buddy	21	F
FBB07	Youth Buddy	20	M
FBB08	Youth Buddy	22	F
FBB09	Youth Buddy	22	M
FBB10	Youth Buddy	22	M
FBH1	Church Leader	40	F
FBH2	Church Leader	40	M
VHW01	Lay Health Worker	46	M
VHW02	Lay Health Worker	45	M
VHW03	Lay Health Worker	51	M
VHW04	Lay Health Worker	42	M
VHW05	Lay Health Worker	45	F
VHW06	Lay Health Worker	45	F
VHW07	Lay Health Worker	49	F
VHW08	Lay Health Worker	42	F
VHW09	Lay Health Worker	51	F
YP1	Young person with lived experience of CMD	19	M
YP2	Young person with lived experience of CMD	22	M
YP3	Young person with lived experience of CMD	22	F
YP4	Young person with lived experience of CMD	21	M
YP5	Young person with lived experience of CMD	17	F
YP6	Young person with lived experience of CMD	23	M
YP7	Young person with lived experience of CMD	24	M
YP8	Young person with lived experience of CMD	21	F
YP9	Young person with lived experience of CMD	21	M
YP10	Young person with lived experience of CMD	24	M
YP11	Young person with lived experience of CMD	22	F

### 2.4 Data collection

Data for this study were collected using a combination of semi-structured in-depth interviews (IDIs) and focus group discussions (FGDs). This triangulated approach captured diverse perspectives from multiple participant groups, including young people, caregivers, and religious leaders, reflecting both rural and urban contexts. In-depth interviews were conducted with young people who had sought mental healthcare, their caregivers, and church leaders. FGDs were held with peer counsellors and lay health workers affiliated with the FB initiative in Bindura rural and Harare. No formal pilot testing of the interview guides was conducted prior to data collection. However, the first two interviews were reviewed by the research team to identify any ambiguous or poorly worded questions, and minor refinements were made to question phrasing before proceeding with the remaining interviews. Each session of the IDI or FGD, lasting 40–60 min, was conducted by a Research Assistant (RA) who is also a co-author (AM) and holds a bachelor’s degree in social sciences. The RA, fluent in both Shona and English, had prior experience in qualitative data collection in mental health topics, psychological first aid, and received ongoing supervision from the principal investigator (RHM). All interviews were audio-recorded, transcribed verbatim and translated from Shona (the local language) into English. Transcripts underwent quality checks, and were pseudo-anonymised to protect participants’ confidentiality. The finalised transcripts were securely stored on a cloud drive, accessible only to the study team in line with laid down role-based data access controls.

### 2.5 Researcher positionality and reflexivity

The research team had prior engagement with the FB through previous mental health projects. This prior relationship facilitated access to programme records and staff. To maintain objectivity, the following practical steps were taken. First, the research team openly acknowledged this prior relationship and discussed how it might shape expectations, noting that familiarity could lead to assumptions and the programme’s value. Second, the interview guides were deliberately designed to ask about both positive and negative experiences, including what did not work or what prevented young people from seeking help. Third, during data analysis, the research team made a conscious effort to return to the raw data (transcripts) regularly rather than relying on memory or prior impressions, ensuring that findings were grounded in what participants actually said. These steps ensured that prior familiarity with the FB did not lead to one-sided data collection or interpretation.

### 2.6 Data analysis

Data for this study were analysed using an inductive thematic approach (48), ensuring analysis remained grounded in participants’ perspectives. All coding and analysis were conducted solely by the principal investigator (RHM) using NVivo software. To ensure rigour despite single-coder analysis, two strategies were employed. First, the research team met weekly to review code groupings and emerging themes, providing ongoing peer scrutiny of analytic decisions. Second, the coding process followed three phases: open coding (line-by-line, generating descriptive codes such as “fear of gossip”), focused coding (collapsing similar codes into broader categories such as “privacy concerns,” and thematic development). The final thematic structure, comprising four primary themes (stigma, distrust, family reputation, and gendered norms) was developed iteratively.

## 3 Findings

The qualitative analysis revealed multiple, interrelated themes shaping young people’s decisions to seek or avoid help for CMDs. These themes illustrate the complex interplay of stigma, family expectations, and social norms, highlighting the social and cultural context of mental health in Zimbabwe.

### 3.1 Stigma

Stigma refers to the negative social attitudes, judgement, and discrimination directed towards individuals experiencing mental distress, as well as internalised fear of being devalued or ostracised by others. Stigma was consistently identified as a major barrier to help-seeking. Young people fear ridicule, social judgement, or public disclosure of their struggles, which discourages them from seeking mental health support. Anticipated negative reactions from peers, families, or institutions create anxiety and silence open discussions about mental health. For instance, a caregiver described the challenges of discussing sensitive health issues within the family and community:

*It is difficult to open up because of fear that everyone will end up knowing their issues. For example if one is suffering from an STI, it is difficult for the young one to open up because they fear people will end up discussing it publicly-*CG2.

When further probed whether they had ever hidden information from the community regarding their child experiencing depression, the caregiver added:

*Yes I once hid it about my child. Knowing that she was HIV positive it was difficult for me even to tell her. I was afraid that if people became aware of my child’s status they will point at my child every time when they meet her saying ‘this one is sick’. You will always be scared to open up because you don’t know how people will react-*CG2.

This quote illustrates intersectional stigma, where HIV status and mental health concerns overlap. The caregiver’s fear of “public pointing” reveals that mental health stigma compounds existing HIV-related stigma. For young people living with both HIV and depression, the anticipated judgement may be twofold: being labelled as “sick” for their HIV status and “weak” or “crazy” for mental distress-creating a double burden that deepens silence and concealment.

Also, young people with lived experiences of mental illness described a profound tension between the need for help and the desire to protect their dignity. One participant noted:

*We might not be able to fully open up because of the community around us and fear as well. Imagine a situation whereby as a young couple you get into a fight and your wife beats you, even going to the police can be hard because they will also laugh at you…. So, it’s very difficult you can keep your issues to yourself even to your death…to protect your dignity*-YP3.

This highlights the deep sense of vulnerability that prevents disclosure, even in situations where support may be available. Similarly, another participant reflected on the impact of fear and shyness on help-seeking.

*I cannot say there is a belief but rather shyness and fear… one cannot be able to tell her story to someone in fear that they can be told that your story has no value or to be laughed at. Mostly its only fear that causes one not to be able to go and open up to anyone-*YP6.

Lay health workers also recognised the impact of stigma on access to mental healthcare:

*Obviously if people discriminate against them, this affects seeking help because their families will not take them to help at the clinic-*VHW05.

Taken together, these findings illustrate that stigma operates not only at the level of overt discrimination but also through internalised fear leading to self-isolation and reluctance to seek social or professional support.

### 3.2 Lack of trust in community and institutional spaces

Distrust refers to a lack of confidence that personal information shared in confidence, whether family, community members, religious leaders, or health providers, will remain private and not be used against the individual or their family. In this study, participants reported distrust in the confidentiality of community and institutional spaces. They described how fears of gossip and mishandling of sensitive information discourage help-seeking. This is not only about social judgement but about whether personal struggles would remain private once disclosed.

Church leaders acknowledged this challenge:

*In some communities they are very reserved and do not want other people knowing their issues so they have trust issues. I’m not sure if you are familiar with the environment of living in an informal housing compound, word moves around fast so they want to keep their issues to themselves and they do not trust easily*-FBH1.

Such caution may stem from prior experiences where confidentiality was breached or norms allowed open discussion of private matters. These fears may extend to counselling services, where individuals worried about being seen or identified:

*Some of them* [young people] *are not sure about how they can come to you without people knowing that they need help ….some do not want to commit to follow-up counselling because they expect a quick-fix and say pray for me and fix my problems-*FBH1.

However, this theme draws more heavily on provider perspectives than youth voices, with only one youth quote directly addressing distrust to corroborate provider accounts. They reported that breaches of confidentiality in faith settings could lead to humiliation:

*If you go with your problems to church, they might end up talking about your problems in front of the church. So young people might not feel comfortable sharing their problems-*YP1.

These accounts demonstrate how mistrust, fear of disclosure, and concerns about confidentiality reinforce a culture of silence around mental health. When individuals believe their struggles may be exposed or misused, they are less likely to seek professional or community-based support.

### 3.3 Family and societal expectations

Family and societal expectations refers to the obligation to protect family honour by concealing mental health struggles, as disclosure is seen as shaming or degrading the standards of the family. In this study, family and societal expectations also emerged as powerful influences on young people’s help-seeking behaviours. Many participants reported that disclosing emotional or psychological difficulties could be seen as reflecting poorly on the family, leading to concealment of problems and avoidance of help. Within this context, maintaining the family’s image often take precedence over addressing individual well-being. As one young person with a lived experience of depression explained:

*Each and every family has their own rules and belief(sic)…, young people can be scared to share their* [mental health] *problems with others in order to protect the family’s reputation. When they share, it will be considered as degrading the standards of the family**-***YP4.

This demonstrates how social expectations about family status and respectability can discourage open discussions about distress. Similarly, another participant described how perceptions of wealth and social class shape responses to mental health concerns:

*Sometimes they will be scared thinking if I go out saying my child has this problem it won’t give us a good picture with how rich we are and the life we live. It might not end well so they will stop looking for help-*YP2.

These pressures are reinforced by prevailing beliefs that strength and endurance are markers of good character. Families that value emotional restraint may interpret help-seeking as a sign of weakness, as noted by a peer counsellor:

*Some families believe they are strong and are not affected by stress. Like in their family history no one suffered from depression or anxiety, so they will question why you are feeling this way, so you will be told ‘you not strong enough’, ‘you need to be strong’-*FBB08.

Lay health workers echoed this view, highlighting that young people often internalise high family expectations and fear disappointing their parents:

*Some young people do not share their problems because their families have high hopes and expectations for them. Let me take an example of a child who is known to be well behaved but has contracted an STI. They won’t feel free to share this with their family because they are known as a well-behaved child-*VHW06.

This quote implies young people may be more honest with lay health workers than their own families. The phrase “wont feel free to share this with their family” suggests trust in providers can exceed family trust, especially when family expectations feel judgemental.

These findings highlight that social status, family reputation, and expectations of resilience can create emotional barriers to help-seeking. Young people may suppress distress to avoid bringing shame upon their families or to preserve an image of strength and stability. Over time, this internalised pressure can reinforce silence around mental health struggles and limits opportunities for early support or intervention.

### 3.4 Gender norms

Gender norms are social rules about how young men and women should behave. For young men, these rules expect them to be tough, hide their emotions, and handle problems alone. For young women, these rules expect them to be calm, obedient, and protect their family’s reputation by not showing distress. Thus, gender norms play a pivotal role in shaping how young people perceive emotional expression and mental health help-seeking. Across discussions, participants emphasised that societal expectations around masculinity and femininity often dictate how distress is expressed or silenced. Boys and young men, in particular, were described as being socialised to suppress emotions and avoid seeking psychological support, as vulnerability is commonly equated with weakness. One community health worker explained:

*Boys are raised to be strong, and are not supposed to show any emotions…they think seeking counselling is a sign of weakness. Because of that, they will not seek help-*CHW2.

This belief reinforces the notion that strength is demonstrated through emotional control rather than openness. As a result, many young men may internalise distress or resort to unhelpful coping mechanisms instead of seeking support. Similarly, social expectations tied to family roles and birth order also shape how young people respond to mental health challenges.

*The society expects the first born child to be someone very responsible, they are required to carry everyone’s burden in the family and if they prove to be emotional and want to talk about their mental health issues, they are viewed as not strong enough and not acting proper to their role as the first born child-*FBB04.

This finding raises whether pressure on first-born children applies equally to males and females. Our data suggest both face heightened expectations, but the consequence differ. First-born males who express distress are seen as failing at masculinity, being “not strong enough.” First-born females are seen as failing after their caregiving role, being “not responsible enough.” Both are punished, but through different gendered norms: stoicism for males, nurturing and composure for females.

For others, expectations often centre on emotional responsibility and composure. Participants highlighted that even when young people attempt to express distress, their feelings are frequently dismissed or minimised within families. As one peer counsellor described:

*Parents don’t expect their children to be stressed….if you tell them you are stressed they will say ‘why are you stressed when you have a roof over your head, you have clothes so why are you sad’…they expect us to be grateful of what we have and not feel stressed-*FBB06.

Such reactions reflect broader cultural belief that young people’s psychological struggles are illegitimate or trivial, further discouraging open communication and help-seeking.

In summary, these excerpts reflect how deeply entrenched gender norms shape the emotional lives of young people. By discouraging emotional disclosure, particularly among males, and reinforcing expectations of resilience and composure, these norms contribute to silence around mental health.

### 3.5 Cross-thematic analysis: intersections and mutual reinforcement

The four themes presented above do not operate independently. As our findings suggest, they may intersect and actively reinforce one another to create a layered ecology of silence. Stigma and distrust mutually reinforce each other: the fear of judgement and the fear of confidentiality breach are not separate barriers but operate as a vicious cycle. When young people anticipate stigma, they become hypervigilant about disclosure, which fuels distrust in institutions. Conversely, when confidentiality is breached or perceived as likely to be breached, this validates stigma by demonstrating that mental health struggles indeed lead to public exposure. The fear of stigma makes trust impossible; the absence of trust makes stigma inevitable.

Gender norms modify family reputation pressures, as the pressure to protect family honour operates differently for young men versus young women. For young men, disclosing mental distress threatens family reputation because it violates masculine norms of strength and stoicism. For young women, disclosing mental distress threatens family reputation because it raises questions about morality, sexual propriety, or marriageability. Thus, the same underlying pressure-protect family honour-produces gender specific manifestations and consequences.

Stigma and family reputation amplify each other. Individual stigma, or the fear of being labelled as mentally ill, is amplified by family-level stakes, as disclosing problems is considered to be against the family’s standards. This means that the cost of disclosure is not merely personal humiliation but collective shame. Young people in this study consistently framed their silence as protective of family, not just themselves. This family level amplification distinguishes collectivist contexts from individualistic settings where stigma operates primary at the individual level.

Finally, gender norms intersect with age and birth order. The pressure on first-born children is itself gendered in practice. While the expectation of responsibility applies to first-born children regardless of gender, the consequences of violating this expectation differ. First born males or females who express emotional distress are seen as failing, but differently. Both are ‘punished’, but through different mechanisms.

Taken together, these intersecting dynamics demonstrate that silence is not merely an individual choice but a socially produced outcome, shaped by the mutual reinforcement of stigma, distrust, gendered expectations, and family honour.

## 4 Discussion

This study has explored the multiple, interrelated sociocultural factors shaping young people’s desire and decisions to seek or avoid help for depression in Zimbabwe. The data point to four primary themes: stigma, mistrust, family expectations and gendered norms. Together, these factors appear to contribute to an environment where mental health problems are actively concealed rather than addressed.

Stigma emerged as a central, multi-faceted barrier to help-seeking. Participants’ profound fear of public judgment and ridicule forbade them from seeking help with CMDs. This aligns directly with the concept of perceived stigma, the belief that one will be devalued and discriminated against by others (21, 49). The anticipation that their mental health struggles would become public knowledge, leading to social ostracisation reflects a rational calculation of social risk, which is a recognised and significant factor in delaying help-seeking behaviour (50). This external fear is frequently internalised, leading to what Goffman (51) termed a ‘spoiled identity’ and what is conceptually understood as internalised stigma (52). The participants’ strategy of ‘self-silencing’ to protect their dignity appears to be a manifestation of this process, encompassing feelings of alienation and a tendency towards social withdrawal to pre-empt rejection.

The powerful account of a caregiver concealing a child’s HIV status for fear of ‘public pointing’ illustrates both enacted discrimination and the anticipatory stigma that arises from it. For the caregiver, sustained hypervigilance and social isolation may increase susceptibility to comorbid anxiety and depression. For the child, secrecy may risk poor treatment adherence and psychosocial developmental delays, representing significant comorbidities that compound the primary HIV diagnosis. This finding suggests how mental health stigma can intersect with other stigmatised conditions to compound marginalisation (53). This, in turn, can perpetuate a cycle where stigma-driven secrecy reinforces the very misconceptions that fuel it, solidifying harmful stereotypes of people with mental illness as dangerous, unpredictable, or culpable for their condition (54). Critically, stigma may cause young people to miss the window for early intervention, allowing mild distress to escalate into more severe difficulties. In Zimbabwe’s resource-scarce system, such delays may contribute to treatable conditions becoming more chronic, potentially further straining overburdened services.

Closely linked to stigma, distrust in community and institutional spaces further restricts help-seeking. In an environment where personal struggles are likely to become public gossip, silence emerges not as a passive condition, but as an active and logical strategy of self-preservation (34, 55). This distrust of both community and institutional settings, including churches and counselling services, was reported as a rational response to the realistic expectation that confidentiality might be breached. This distrust challenges community-based mental health models, which rely on perceived safety and confidentiality (56). The Friendship Bench has been scaled nationally, yet young people rarely access it due to privacy concerns. Distrust may help explain why services remain unused even when available. These findings align with the principle that trust is a prerequisite for effective care. However, our data suggests that for young people in Zimbabwe, mistrust is not simply absence of trust but an active, learned response to anticipated breaches of confidentiality and feared social consequences. Participants described how past experiences of gossip or observed breaches of confidentiality shaped their current reluctance to seek help. This finding suggests that community-based models may need to actively build trust and demonstrate confidentiality consistently, rather than assuming that service availability alone will lead to uptake (57).

Additionally, the imperative to protect family reputation emerged as another sociocultural barrier to help-seeking. In the context of Zimbabwe’s collectivist culture, an individual’s mental health condition was viewed by participants as not a private matter but a potential stain on the entire family’s social standing and honour (58, 59). Young people reported concealing their problems to avoid degrading the standards of the family and to maintain a façade of success and stability. This reported pressure creates a dynamic where the family, which should be the core source of support, becomes a reason of concealment, which is consistent with prior literature in low-resource settings. This phenomenon is rooted in collectivist values that prioritise group cohesion and familial harmony (60, 61), making help-seeking a family-centred decision (62). When a family perceives mental illness as a threat to its social standing, some participants reported that the collective response is to conceal it (63). This collectivist pressure may be intensified by honour-culture norms, where a family’s status depends on its reputation for strength. Mental illness, stereotyped as a sign of weakness (64), is a direct threat to this reputation, further fuelling a cycle of silence (65–67). Based on these findings, interventions aimed at increasing help-seeking in these contexts may need to address both access to care and the stigma associated with mental illness at the familial and community levels.

Alongside these powerful sociocultural factors, gender norms emerged as a critical and distinct barrier, socially engineering specific help-seeking pathways for young people in Zimbabwe. The observation that “boys are raised to be strong and are not supposed to show any emotions” provides a clear example of hegemonic masculinity in action. This concept, as defined by Connell and Messerchmidt (68), refers to a dominant form of masculinity that upholds patriarchal power and is characterised by emotional stoicism, self-reliance and control, traits historically shaped in Africa by socio-political forces (38–40). For a young man, seeking mental health support is thus not merely a health-seeking behaviour, but a direct violation to this core masculine ideal, contradicting norms of self-reliance and invulnerability (69, 70). This appears to create a barrier, consistent with previous findings that such conformity leads to lower help-seeking and increased self-management (71). The fear of being seen as weak may contribute to gender role conflict (72), pathologising vulnerability and equating emotional expression with a failure of character. For young women, while the data is less explicit, gendered expectations also shape their experience in ways that constrain help-seeking. The societal pressure to be compliant and the specific reputational risks associated with female mental health, potentially affecting perceptions of reliability or marriageability, may create different set of constraints rooted in idealised femininity (73).

Gender also interacts with birth order. First-born children may face higher expectations for responsibility and behaviour (74, 75). Our findings show these expectations are gendered in their consequences: first-born males who express distress are seen as failing at masculinity (“not strong enough”), while first-born females are seen as failing at caregiving (“not responsible enough”). These norms appear to be reinforced by generational beliefs that dismiss youth stress (76). In the Zimbabwean context, the public health consequences are unequal: young men may delay until self-harm or substance dependence, while young women may remain trapped in silence while distress worsens. With the country’s high youth unemployment rate, CMDs may undermine young people’s capacity to work, creating a feedback loop where mental distress perpetuates poverty across generations. This underscores the critical need for gender-transformative interventions that address these specific, culturally constructed barriers to mental healthcare.

Importantly, as this study identified barriers rather than testing interventions, the following significant implications are derived from participants’ reported experiences and are intended to be generative rather than prescriptive. For mental health programming, interventions could consider adopting multi-level approaches that simultaneously address these interconnected barriers. Community-based programmes like the Friendship Bench could develop components to counter stigma and build trust. Given the influence of family reputation, interventions might incorporate family engagement strategies that reposition help-seeking as a strength-based action that preserves family well-being rather than threatening it. The findings on gender norms suggest the value of gender-responsive programming that specifically challenges hegemonic masculinity while creating safe spaces for young men to express vulnerability, and that addresses the unique reputational risks faced by young women.

At the policy level, these results suggest that improving the availability of mental health services alone may be insufficient. National mental health strategies could benefit from supporting community-led anti-stigma campaigns and gender-transformative programming. Policies might also promote mental health literacy initiatives targeting families and community leaders. The Zimbabwean government’s current mental health investment, already severely underfunded, may need to address not only workforce shortages but also the sociocultural barriers that prevent young people from accessing the existing services.

For research purposes, future research could focus on developing and testing integrated intervention models based on barriers identified here. Implementation science could help adapt evidence-based interventions like the FB for youth populations. Without such efforts, the treatment gap for young Zimbabweans may remain, in part, because pathways to care remain blocked by the sociocultural barriers identified in this study.

### 4.1 Strengths and limitations

This study has several notable strengths alongside some limitations that should be acknowledged. A primary strength is the methodological rigour achieved through a qualitative design and triangulation of data sources. By gathering perspectives from young people with lived experience, their caregivers, lay health workers, peer counsellors, and church leaders, we obtained rich, multidimensional narratives on sociocultural barriers influencing help-seeking for CMDs. Furthermore, the diverse geographical sampling across urban Harare and rural Bindura captures a wide spectrum of sociocultural contexts, enhancing the transferability of the findings to similar settings. The use of an inductive analytical approach ensured that the themes were firmly grounded in the participants’ own experiences and cultural realities, rather than being constrained by pre-existing frameworks.

Despite these strengths, certain limitations exist. First, and most importantly, all youth participants were treatment engaged, having received care through the FB. The perspectives of young people who avoid help-seeking entirely due to sociocultural barriers are therefore missing, meaning the findings likely represent those who successfully navigated initial help-seeking rather than those who remained completely silent. Second, the samples of caregivers (*n* = 2) and religious (*n* = 2) were very small, and saturation was not achieved for these groups. However, their insights are illustrative rather than exhaustive. Third, social desirability bias may be present, as participants may have underreported socially unacceptable attitudes or behaviours. Fourth, the cross-sectional design provides a snapshot and cannot trace how barriers evolve over time. Finally, this study did not conduct member checking. While strategies such as team reviews were employed, the absence of participant validation means findings reflect the research team’s analytical perspective rather than participant-confirmed interpretations.

## 5 Conclusion

In Zimbabwe, young people’s reluctance to seek help for CMDs is a rational response to stigma, distrust, family reputation concerns, and restrictive gender norms. As our findings suggest, these forces make concealment safer than disclosure. To interrupt this cycle, three actions are warranted. First, Zimbabwe’s health authorities should establish confidential, youth-friendly mental health entry points in non-clinical settings such as schools, community centres, and sports clubs, removing the privacy barriers associated with formal clinics. Second, anti-stigma interventions should target families and community leaders directly, not young people alone, using existing community gatherings to reframe mental health help-seeking as compatible with family honour and strength. Third, policymakers should mandate privacy protections for young people’s mental health records within primary care and allocate dedicated funding for community-led awareness campaigns that engage trusted local figures. Addressing these interconnected barriers can help Zimbabwean youth seek care without sacrificing social standing, family honour or gendered identity.

Participants were fully informed about the study objectives, procedures, potential risks and benefits, and their rights, including the option to participate voluntarily and to withdraw at any time, without being questioned for withdrawing. Confidentiality was ensured through the use of unique study ID numbers and secure, password-protected data storage accessible only to the research team. To address potential distress, the RA was trained in psychological first aid. If a minor disclosed suicidal ideation or risk of harm, the protocol required pausing the interview, offering emotional support, conducting a safety assessment, and notifying a safeguarding lead who would conduct the FB staff for follow-up care. Additionally, parents would be notified only if imminent risk existed. However, no such disclosures occurred. Reimbursement of USD10 covered transport (typical return cost USD3-5) and time. This amount was approved by the ethics committee (MRCZ) as it offsets expenses without constituting undue inducement. Participation was strictly voluntary, with non-coercion or undue influence, and all interactions were conducted to protect participants’ privacy and dignity.

## Data Availability

The raw data supporting the conclusions of this article will be made available by the authors, without undue reservation.
